# Development of an ligase chain reaction–fluorescence–SERS multimodal biosensing workflow for ultrasensitive detection and single-base discrimination of *KRAS* ctDNA

**DOI:** 10.1007/s00604-026-08168-3

**Published:** 2026-06-13

**Authors:** Sohila Mostafa, Dilek Kanarya, Khwanchai Tantiwanichapan, Raju Botta, Pitak Eiamchai, Pacharamon Somboonsaksri, Nimet Yıldırım Tirgil

**Affiliations:** 1 Department of Biomedical Engineering, Ankara Yıldırım Beyazıt University, Ankara, Türkiye; 2 Institute of Biomedical Engineering - MIBE, Department of Electrical Engineering, TUM School of Computation, Information and Technology, Technical University of Munich, Munich, 80333 Germany; 3 National Electronics and Computer Technology Center (NECTEC), National Science and Technology Development Agency (NSTDA), 112, Khlong Nueng, Khlong Luang, Pathum Thani 12120 Thailand; 4 Department of Metallurgy and Materials Engineering, Ankara Yıldırım Beyazıt University, Ayvalı Mh. Takdir Cad.150 Sk. No:5 Etlik-Kecioren, Ankara, 06010 Türkiye

**Keywords:** Circulating tumor DNA (ctDNA), Ligase chain reaction (LCR), Fluorescence monitoring, Surface-enhanced Raman scattering (SERS), Biosensor

## Abstract

A dual-modality biosensing platform integrating ligase chain reaction (LCR), fluorescence monitoring, and surface-enhanced Raman scattering (SERS) was developed. The LCR step ensures high-fidelity enzymatic amplification, enabling precise discrimination of single-base mismatches at the KRAS locus. Real-time fluorescence using 20 nm gold magnetic nanoparticles provides rapid quantitative evaluation, while SERS analysis on nanostructured OnSpec-Lite bimetallic chips offers molecular-fingerprint confirmation. Under optimized conditions (30 cycles, 60 °C), the biosensor achieved a calculated limit of detection (LOD) of 1.03 fM for SERS and 11.94 fM for fluorescence detection, with corresponding limits of quantification (LOQ) of 3.12 fM and 36.17 fM, respectively. Derived from the linear regression of the SERS calibration curve (y = 73.77x + 871.1; R2 = 0.9617), a linear concentration range of 1 pM to 1000 nM was obtained. Meanwhile, the fluorescence calibration yielded a linear range of 100 pM to 1000 nM (y = 0.069x + 4.848; R2 = 0.8705). Experimentally, the lowest validated ctDNA concentrations detected were 1 pM for SERS and 100 pM for fluorescence. Both detection systems demonstrated high reproducibility, with relative standard deviations (RSDs) below 10%. Furthermore, the platforms maintained analytical robustness in 1/10 and 1/5 serum dilutions, successfully mitigating matrix interference while preserving distinctive Raman signatures. This study introduces a proof-of-concept analytical framework that bridges the high specificity of enzymatic ligation with the ultra-sensitive transduction of nanoplasmonic detection. By combining real-time optical quantification with confirmatory vibrational spectroscopy, the system minimizes false positives and provides cross-validated results essential for high-stakes diagnostics.

**Graphical abstract:**

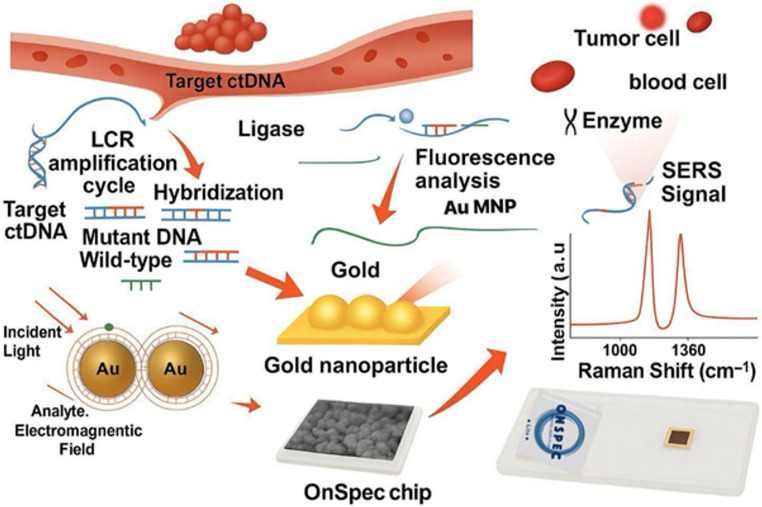

**Supplementary Information:**

The online version contains supplementary material available at 10.1007/s00604-026-08168-3.

## Introduction

Cancer remains a leading cause of morbidity and mortality worldwide. Early and accurate detection is a decisive determinant of treatment outcome, as survival improves markedly when malignancies are identified at an early stage [1]. Conventional histopathological tissue biopsies, although clinically established, are invasive, subject to sampling bias arising from tumor heterogeneity, and unsuitable for longitudinal monitoring [2]. They also impose logistical burdens and depend on chemical fixatives, which limit their routine use. Consequently, liquid biopsy has emerged as a transformative alternative, enabling non-invasive genotyping and disease surveillance by analyzing tumor-derived analytes in blood, among which circulating tumor DNA (ctDNA) is particularly informative. ctDNA fragments originate from apoptosis, necrosis, or active secretion and carry tumor-specific genetic and epigenetic signatures, including point mutations, copy-number variations, and methylation patterns that mirror the molecular landscape of the primary or metastatic lesion [3]. Despite this diagnostic promise, ctDNA usually represents less than 1% of total cell-free DNA and exhibits a short circulation half-life of only a few hours, necessitating analytical systems that achieve both high sensitivity and single-nucleotide specificity [3, 4]. However, reliable ctDNA detection also requires overcoming additional analytical challenges, including low variant allele fractions, DNA fragmentation, matrix interference from circulating biomolecules, and pre-analytical variability associated with plasma processing [5].

Over the past decades, the Polymerase Chain Reaction (PCR) and its quantitative derivatives (qPCR and dPCR) have served as fundamental tools for DNA amplification and mutation analysis [6]. While reliable, these methods often require complex thermocycling instruments, long reaction times, and are susceptible to primer-dimer formation and non-specific amplification, leading to false positives. Moreover, the dependence of PCR on enzymatic polymerization limits its ability to discriminate single-base substitutions within short ctDNA fragments. Alternative approaches, including the Amplification Refractory Mutation System (ARMS) and Beads, Emulsion, Amplification, and Magnetics (BEAMing), have improved rare-allele detection but remain constrained by multi-step protocols and high operational cost [7, 8]. To overcome these limitations, the Ligase Chain Reaction (LCR) employs an enzymatic amplification strategy that leverages the exceptional fidelity of thermostable DNA ligases [9, 10]. In LCR, adjacent oligonucleotide probes hybridize precisely to a target DNA strand, and ligation occurs only when the probe junctions are fully complementary [11]. This strict base-pairing requirement enables discrimination of single-nucleotide differences between mutant and wild-type sequences [6, 12]. Through cyclic denaturation and ligation, LCR yields exponential amplification from trace amounts of input. Its simple probe design and compatibility with fluorescence or spectroscopic readouts make it an attractive choice for integration into multimodal biosensing systems. Beyond spectroscopic biosensing, ligation-based mutation-detection strategies have also been integrated with alternative signal transduction mechanisms, including acoustic sensing, electrochemical detection, and fluorescence resonance energy transfer, demonstrating the versatility of LCR for highly selective single-nucleotide variant analysis [13].

Fluorescence detection naturally complements LCR by providing a rapid, quantitative means to monitor reaction kinetics in real time. Labeled probes or intercalating dyes enable dynamic optimization of ligation temperature, enzyme concentration, and cycle number, improving both amplification efficiency and analytical reproducibility [14]. In parallel, Surface-Enhanced Raman Scattering (SERS) provides molecular fingerprint information with remarkable sensitivity and chemical specificity. Recent advances in SERS-based liquid biopsy platforms include label-free detection systems, nanoparticle nanotag strategies, magnetic bead enrichment, microfluidic integration, and paper-based sensing formats; however, challenges related to spectral reproducibility, matrix interference, and inter-laboratory standardization remain active areas of research [15, 16]. The excitation of localized surface plasmon resonances in metallic nanostructures enhances Raman scattering by several orders of magnitude [12, 17]. Gold-based nanostructured substrates—particularly NECTEC’s gold-coated OnSpec-Lite chips—offer high signal reproducibility and stability, positioning them as reliable SERS candidates for diagnostic use [18].

By integrating LCR, fluorescence, and SERS within a unified workflow, it becomes possible to combine the molecular-recognition precision of enzymatic ligation with the quantitative power of optical detection and the chemical specificity of vibrational spectroscopy. While the proposed detection steps are executed sequentially rather than simultaneously, our platform is explicitly designed as a unified triple-module workflow that uses the same amplified sample for both readouts. In this sequential workflow, fluorescence provides real-time quantitative monitoring, while SERS provides molecular-fingerprint confirmation. Such hybrid systems overcome the limitations of single-modality assays, offering cross-validated results that minimize false positives, are highly sensitive, and reproducible ctDNA detection [19, 20].

## Materials and methods

### Materials and reagents

All DNA oligonucleotides, including mutant, wild-type, and probe sequences (Table 1) were synthesized by ABclonal Biotechnology and dissolved in nuclease-free water to a working concentration of 10 µM. Additional reagents included T4 DNA ligase (Cat. No. M0202S, New England BioLabs Inc.), 10× Ligase Reaction Buffer (Cat. No. B0202S, New England BioLabs Inc.), and Tris–EDTA (TE) buffer (10 mM Tris–HCl, 1 mM EDTA, pH 8.0) (Cat. No. TEB204, BioShop Canada Inc.). Phosphate-buffered saline (PBS, 10 mM, pH 7.4), UltraPure™ Agarose (Cat. No. 16500500, Invitrogen), and Ethidium Bromide (Cat. No. RM21501, ABclonal) were used for DNA visualization. Gold-based SERS substrates (OnSpec LITE; Opto-Electrochemical Sensing Research Team, National Electronics and Computer Technology, Thailand) were utilized as nanoplasmonic platforms for Raman spectroscopic measurements [21]. Gold-coated magnetic particles (Au MNPs; 20 nm and 50 nm) used as fluorescence reporters were purchased from CD Bioparticles, NY/USA.

**Table 1:** DNA oligonucleotides used in this study

Name	Sequence (5′ → 3′)	Description
Target ctDNA	AAGCTTGCTAGCCGAAAATATAAACTTGTGGTAGTTGGAGCTGGTGGCGTAGGCAAGAGTGCCTAAGGTGCAATCCGTA	Wild-type ctDNA fragment corresponding to the KRAS locus
1-base mutant DNA (C1)	AAGCTTGCTAGCCGAAAATATAAACTTGTGGTAGTTGGAGCTGATGGCGTAGGCAAGAGTGCCTAAGGTGCAATCCGTA	Single-nucleotide variant for selectivity validation
4-base mutant DNA (C2)	AAGCTTGCTAGCCGAAAATATAAACTTGTGGTAGTTGGAGCTGATGGCGTAAACAAGGGTGCCTAAGGTGCAATCCGTA	Four-base mutant representing multi-site alteration
Common primer	ACCATCAACCTCGAC	Universal primer used in the LCR cycle
Discriminating primer	CACCGCATCCGTTCT (5′-FAM)	FAM-labeled primer enabling fluorescence detection
Probe DNA	Thiol-TGGTAGTTGGAGCTG	Thiolated capture probe immobilized on Au substrate for SERS analysis

### Instruments

Thermal cycling for LCR was performed using a LongGene A300 Fast Thermal Cycler to ensure accurate temperature control. Fluorescence intensities were measured with a microplate reader (Vilber Lourmat, Varioskan, Thermo Scientifics Inc.) at 495 nm excitation and 520 nm emission. Agarose gel images were captured under 302 nm UV illumination with a gel documentation system (Quantum-ST4 1100/26 M). Raman spectra were obtained with a JASCO NRS-4500 laser Raman spectrometer equipped with a 532 nm laser, 10× objective, 1.8 mW laser power at the sample (measured at the 10× objective), 100 accumulations × 0.01 s exposure per accumulation (1 s total integration per spectrum).All instruments were calibrated before each run, and all measurements were conducted at ambient room temperature (RT).

### Fabrication of Al/Au SERS sensors

The detailed fabrication process of the SERS substrates used in this study has been reported previously [18]. A brief summary is provided here for completeness. The Al/Au SERS sensors, marketed as OnSpec-Lite SERS chips, were fabricated using a two‑step procedure that combined laser‑induced micro‑textured aluminum (Al) and gold nanoparticle (AuNP) deposition [19, 22]. The process was engineered to create high‑density plasmonic “hot spots” on a stable metallic platform, as clearly depicted in Figure S1.

In the first stage, high‑purity Al sheets were cleaned thoroughly with ethanol and deionized water to remove organic contaminants. The cleaned surfaces were then micro-textured using a 1064 nm laser marking system (Smart Mark, Photonics Science Co., Ltd., Thailand), which produced controlled microscale features through localized melting followed by rapid re‑solidification. Laser engraving was carried out at 12 W power, 20 μm fill spacing, and a 30 kHz repetition rate, optimized towards consistent groove and ridge patterns.

In the second stage, Au nanoparticles were deposited onto the laser‑textured Al substrates using a custom-designed, laboratory‑assembled, single‑cathode PVD system (DC magnetron sputtering). The base chamber was evacuated to 5.0 × 10⁻⁶ mbar, and the deposition was then performed at 3.0 × 10⁻³ mbar, 0.1 A cathode current, and 90 W DC power for 270 s. Under these conditions, Au nanoparticles nucleated across the engraved ridges and valleys, forming a uniform thin film with nanoscale roughness. After deposition, representative SERS samples were examined using field‑emission scanning electron microscopy (FE‑SEM) to confirm homogeneous distributions of AuNPs on the Al surface. The average AuNP size was ~ 50 ± 15 nm, with interparticle gaps ranging from 20 to 40 nm serving as electromagnetic “hot spots” [17, 18]. The samples were further validated using Paraquat as a standard Raman probe, with at least 4 major Raman bands at 840, 1190, 1300, and 1650 cm⁻¹, to achieve relative standard deviation (RSD) values below 10%. Finaly, the obtained SERS substrates were diced into 5 × 5 mm² chips, mounted on glass slides for ease of handling, and sealed in metalized vacuum packages to prevent contamination prior to use.

### Ligase chain reaction (LCR)

The LCR served as the principal amplification mechanism for the selective detection of mutant and wild-type ctDNA sequences with single-base accuracy [9, 10]. Each 20 µL reaction contained ligase buffer, 5 nM of both primers, and 2 µL of T4 DNA ligase (200 000 U mL⁻¹). Discriminating primers were FAM-labeled to generate a fluorescence signal only when successful ligation occurred, ensuring specificity. The optimized parameters and their experimentally determined values are listed in Table S1.

Thermal cycling consisted of denaturation at 94 °C for 30 s followed by hybridization/ligation at 60 °C for 1 min per cycle. These steps enabled precise probe annealing and covalent joining, resulting in exponential amplification of the correct sequence. Reaction parameters, including hybridization temperature, enzyme concentration, cycle number, and DNA concentration, were optimized to achieve maximum signal intensity and reproducibility (Table S1). Control reactions were performed with wild-type DNA, a no-template control, and a fully mismatched specificity, and a ligase-free reaction mixture to confirm that signal generation depended on enzymatic ligation during the LCR process [14].

### Gel electrophoresis and fluorescence analysis

Agarose gel electrophoresis was carried out to validate LCR amplification. A 5% gel was prepared in 1× TE buffer containing ethidium bromide (0.5 µg mL⁻¹) and run at 70 V for 45 min [23]. DNA bands were visualized using a UV transilluminator. The expected product (~ 72 bp) was observed, confirming specific amplification under optimized conditions.

Fluorescence quantification was performed using Au MNP [24] conjugates of two different sizes, 20 nm and 50 nm, each functionalized with thiolated DNA primers. Initially, the Au MNP–primer conjugates were incubated for 1 h to allow complete surface functionalization, followed by washing with phosphate-buffered saline (PBS) to remove any unbound primers. Subsequently, the prepared conjugates were hybridized to either the target or the amplified DNA at concentrations ranging from 1000 nM to 0.1 nM. The hybridization was performed at 37 °C for 1 h to ensure specific and efficient binding between complementary DNA strands. After hybridization, the samples were washed again with PBS to remove nonspecifically bound DNA, then resuspended in buffer for fluorescence measurement. Each concentration point was measured in three independent LCR reactions (*n* = 3); fluorescence intensity values reported in the calibration curve represent the mean of these triplicates. Fluorescence emission was recorded using excitation and emission wavelengths of λ_ex_ = 495 nm and λ_em_ = 520 nm, respectively. Comparative analysis revealed that the 20 nm Au MNP conjugates generated stronger fluorescence signals than the 50 nm counterparts, likely due to enhanced nanoparticle dispersion and reduced self-quenching effects [25]. Moreover, the fluorescence intensity was inversely proportional to the target DNA concentration, demonstrating the assay’s high sensitivity and reliability for quantitative detection.

### SERS analysis for ctDNA detection

**Fig. 1:**
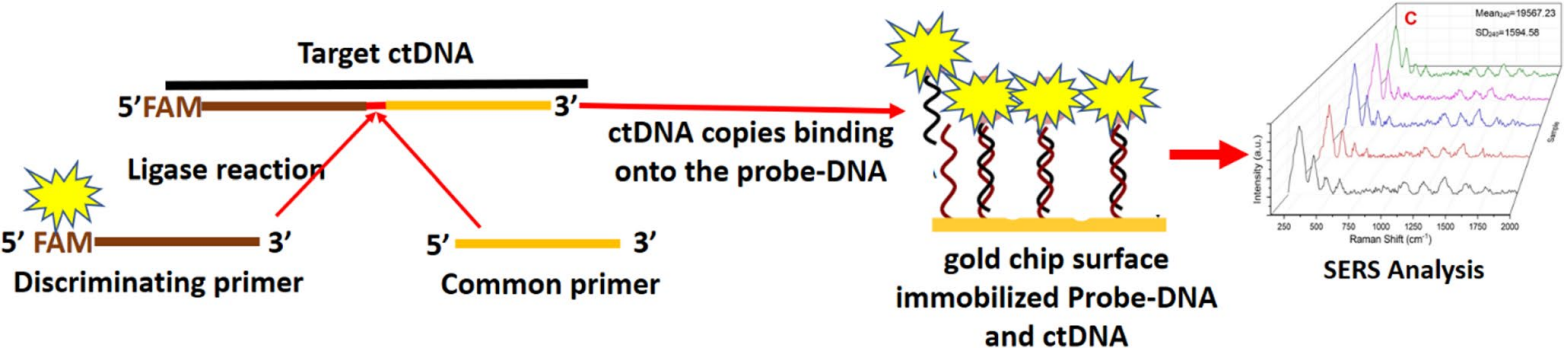
Schematic representation of the SERS-based biosensing strategy for ctDNA detection

SERS chips were cleaned with deionized water and dried under a stream of nitrogen. Each chip was incubated with 10 µL of 10 µM thiolated probe DNA for 45 min at RT to form a self-assembled monolayer via Au–S bonding. After incubation, the substrates were washed three times with 1× TE buffer to remove unbound probes. These washing steps were designed to minimize nonspecific adsorption of free DNA molecules onto the nanostructured surface, ensuring that the measured Raman signals originated predominantly from specifically hybridized probe–target complexes. The samples were then air-dried at room temperature under dust-free, controlled conditions. The functionalized chips were then incubated with 10 µL of target ctDNA or LCR products for 45 min at RT, washed again using the same procedure, and air-dried before Raman analysis. In our sequential dual-readout workflow, the same analyte samples, i.e., the LCR-amplified products, are used for both detection modalities: fluorescence quantification is first performed in solution using Au magnetic nanoparticles, followed by deposition of the same amplicons onto the SERS substrate for Raman spectral confirmation. Specifically, the successfully ligated products, comprising the FAM-labeled discriminating primer and the common primer, first bind to the 20 nm Au magnetic nanoparticles for solution-phase fluorescence quantification. Subsequently, these same amplicons are drop-cast onto the solid OnSpec-Lite chip, where they hybridize with the immobilized thiolated probe, bringing the FAM reporter into close proximity with the plasmonic surface for localized SERS detection. The principle of the developed sensing platform is summarized in Fig. 1.

Specificity was assessed using mutant ctDNA, wild-type DNA, fully mismatched DNA, non-amplified target DNA, and blank control to verify that detectable signals originated from LCR amplification rather than direct adsorption of DNA on the substrate. Raman spectra were acquired using a 532 nm excitation laser (1.8 mW at the sample, 10× objective, 100 accumulations × 0.01 s exposure per accumulation), a configuration selected to provide strong Raman responses while minimizing local heating or photobleaching. For each sample, spectra were collected from ten randomly selected points within the central region of the drop-cast area, ensuring measurements were obtained from zones with uniform DNA–probe coverage and consistent plasmonic activity. Mutant ctDNA samples exhibited distinct peaks corresponding to nucleobase vibrations (~ 730, 1002, 1337 cm⁻¹) and the FAM label, while the control groups showed only weak background signals. These results verified accurate single-base discrimination and confirmed the analytical reliability of the LCR–SERS hybrid biosensor [20, 26].

## Results

### Optimization of LCR reaction conditions

**Fig. 2:**
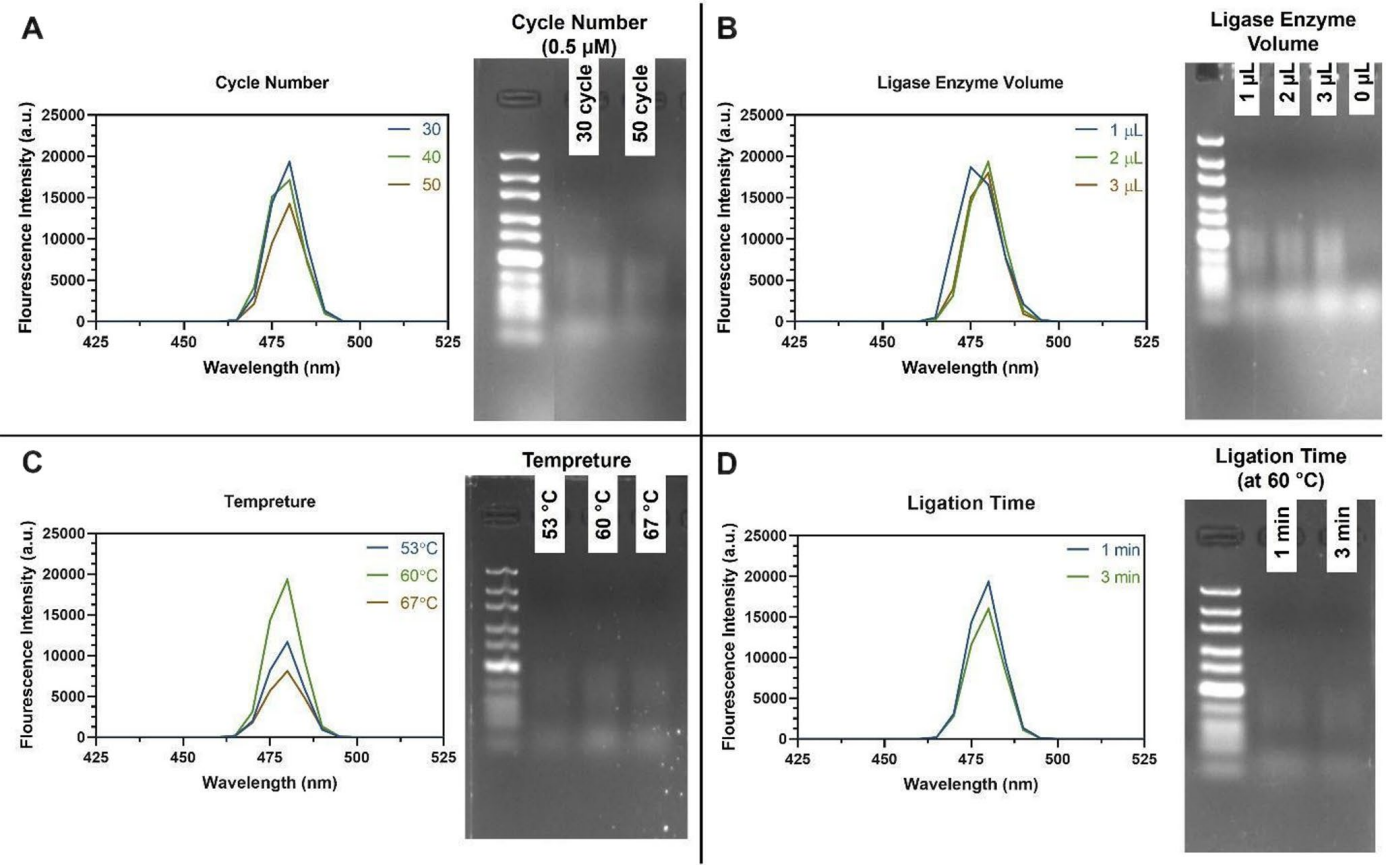
Optimization of the ligase chain reaction (LCR) parameters evaluated through fluorescence intensity and agarose gel electrophoresis. (**A**) Variation in the number of amplification cycles (30–50 cycles). (**B**) Effect of ligase enzyme volume (1–3 µL). (**C**) Influence of ligation temperature. (**D**) Optimization of ligation time per cycle

Optimization of the LCR parameters was conducted to maximize amplification efficiency while minimizing background noise. Four reaction variables, cycle number, enzyme concentration, ligation temperature, and incubation time (see Table S1 were systematically evaluated through both fluorescence intensity measurements and agarose gel electrophoresis analysis (Fig. 2). This dual evaluation enabled a comprehensive assessment of amplification performance, product yield, and reaction specificity under each tested condition.

Fluorescence analysis demonstrated that 30 cycles provided the highest and most reliable signal, whereas extending the reaction to 50 cycles led to a gradual decline in fluorescence due to the accumulation of nonspecific amplification products. Gel electrophoresis supported these observations, showing sharp, well-defined bands at 30 cycles and faint nonspecific smearing at 50 cycles. Therefore, 30 cycles were identified as the optimal balance between amplification efficiency and assay duration (Fig. 2A). Optimizing the ligase volume showed that 2 µL of ligase produced the brightest bands and strongest fluorescence signals, whereas excessive enzyme usage (3 µL) led to partial over-ligation and band diffusion (Fig. 2B). Temperature optimization showed maximum ligation efficiency at 60 °C, with reactions at 53 °C showing incomplete hybridization, while those at 67 °C exhibited diminished ligase activity (Fig. 2C). Comparison of ligation times between 1 and 3 min revealed similar amplification performance, indicating that the enzymatic ligation reached completion within one min, thereby supporting faster reaction cycles without compromising yield (Fig. 2D).

### Fluorescence validation of amplification efficiency

Fluorescence analysis using Au MNPs was conducted to validate the analytical performance of the optimized LCR system, including sensitivity, selectivity, and matrix compatibility. Two nanoparticle sizes, 20 nm and 50 nm were initially evaluated as fluorescence probes functionalized with thiolated primers. As shown in Figure S2, the 20 nm Au MNP conjugates produced significantly stronger fluorescence signals than the 50 nm counterparts, likely due to enhanced nanoparticle dispersion and reduced self-quenching, thereby providing superior signal intensity and assay sensitivity. Based on these results, the 20 nm Au MNP system was employed for all subsequent analyses.

**Fig. 3:**
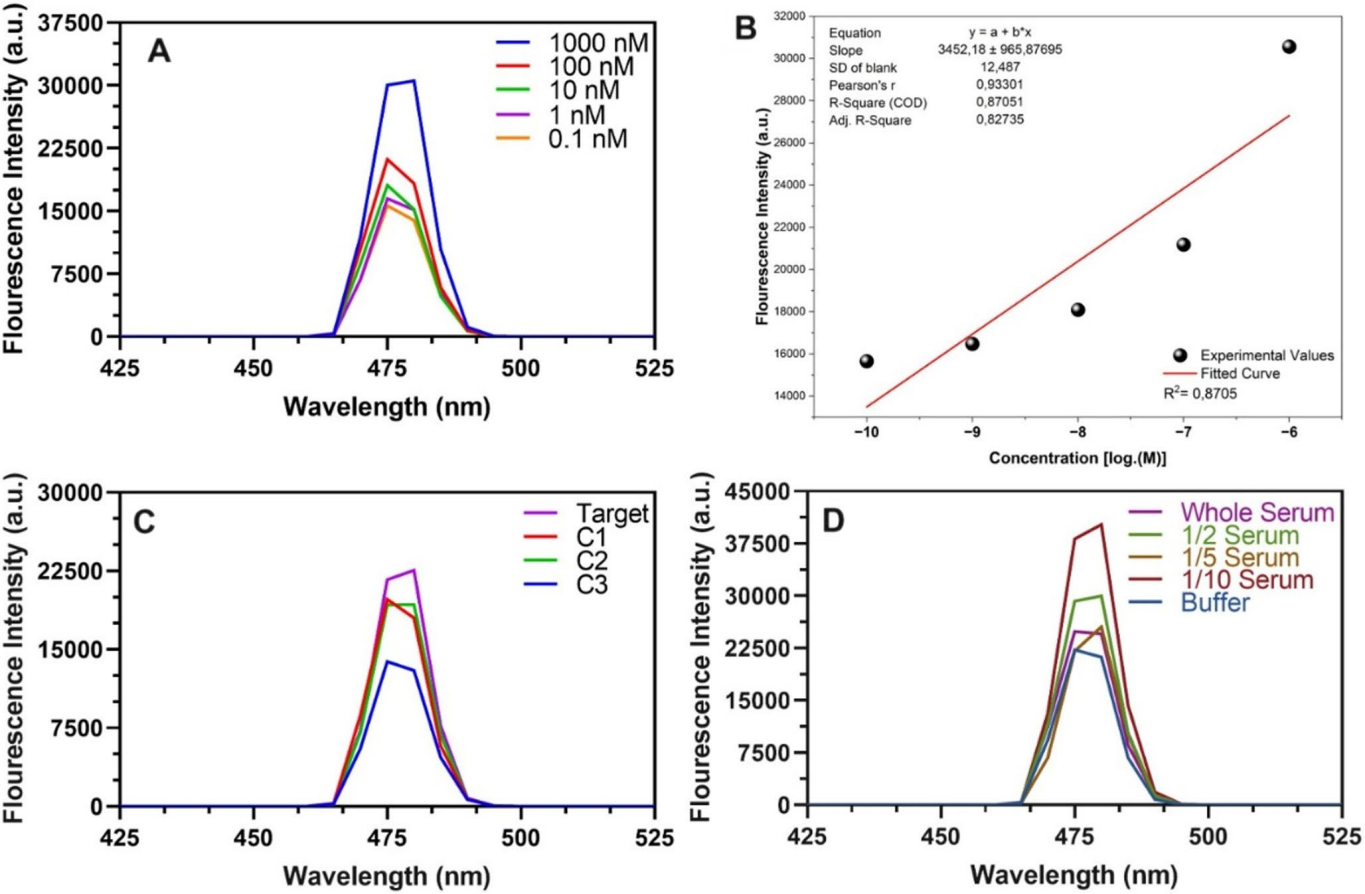
Fluorescence analysis of the optimized Au MNP (20 nm)–based LCR system for ctDNA detection. (**A**) Emission spectrum of LCR products at target DNA concentrations ranging from 1000 nM to 0.1 nM, with a detectable fluorescence signal observed down to 0.1 nM (100 pM). (**B**) Calibration curve of fluorescence intensity versus the logarithm of target concentration (log [M]) across the linear range of 1000 nM to 100 pM; the linear fit is described by y = 0.069x + 4.848 (R² = 0.8705). (**C**) Specificity analysis comparing perfectly matched target and mismatched control sequences (C1, C2, and C3). (**D**) Fluorescence response in serial serum dilutions (whole, 1/2, 1/5, and 1/10) relative to buffer, confirming matrix tolerance and analytical robustness of the Au MNP–based detection system

As illustrated in Fig. 3A, the fluorescence emission intensity of the FAM-labeled LCR products decreased proportionally with target DNA concentration across the range of 1000 nM to 0.1 nM, demonstrating a clear concentration-dependent response. Notably, the lowest tested concentration (0.1 nM, equivalent to 100 pM) still produced a detectable fluorescence signal above the assay background, indicating the high sensitivity of the fluorescence readout for monitoring LCR amplification. The calibration curve (Fig. 3B) showed a linear relationship between fluorescence intensity and the logarithm of DNA concentration over the range of 1000 nM to 0.1 nM (100 pM), described by the equation y = 0.069x + 4.848 (R² = 0.8705), confirming the quantitative reliability of the assay. Specificity evaluation (Fig. 3C) showed that the perfectly matched target produced a significantly stronger fluorescence signal than three mismatched control sequences (C1, C2, and C3), confirming the high selectivity of the LCR amplification and fluorescence detection system for single-base mutation discrimination. In the proposed multimodal sensing workflow, fluorescence primarily serves as a rapid quantitative indicator of LCR amplification efficiency prior to molecular confirmation by SERS analysis. Furthermore, analysis of biological matrices (Fig. 3D) showed that fluorescence intensity gradually decreased with increasing serum content, due to optical and matrix interference. Nevertheless, distinct emission peaks remained detectable at 1/10, 1/5, and 1/2 serum dilutions, indicating that the 20 nm Au MNP–based LCR system maintains high robustness and reliable performance under physiologically relevant conditions.

### SERS confirmation of ctDNA detection

**Fig. 4:**
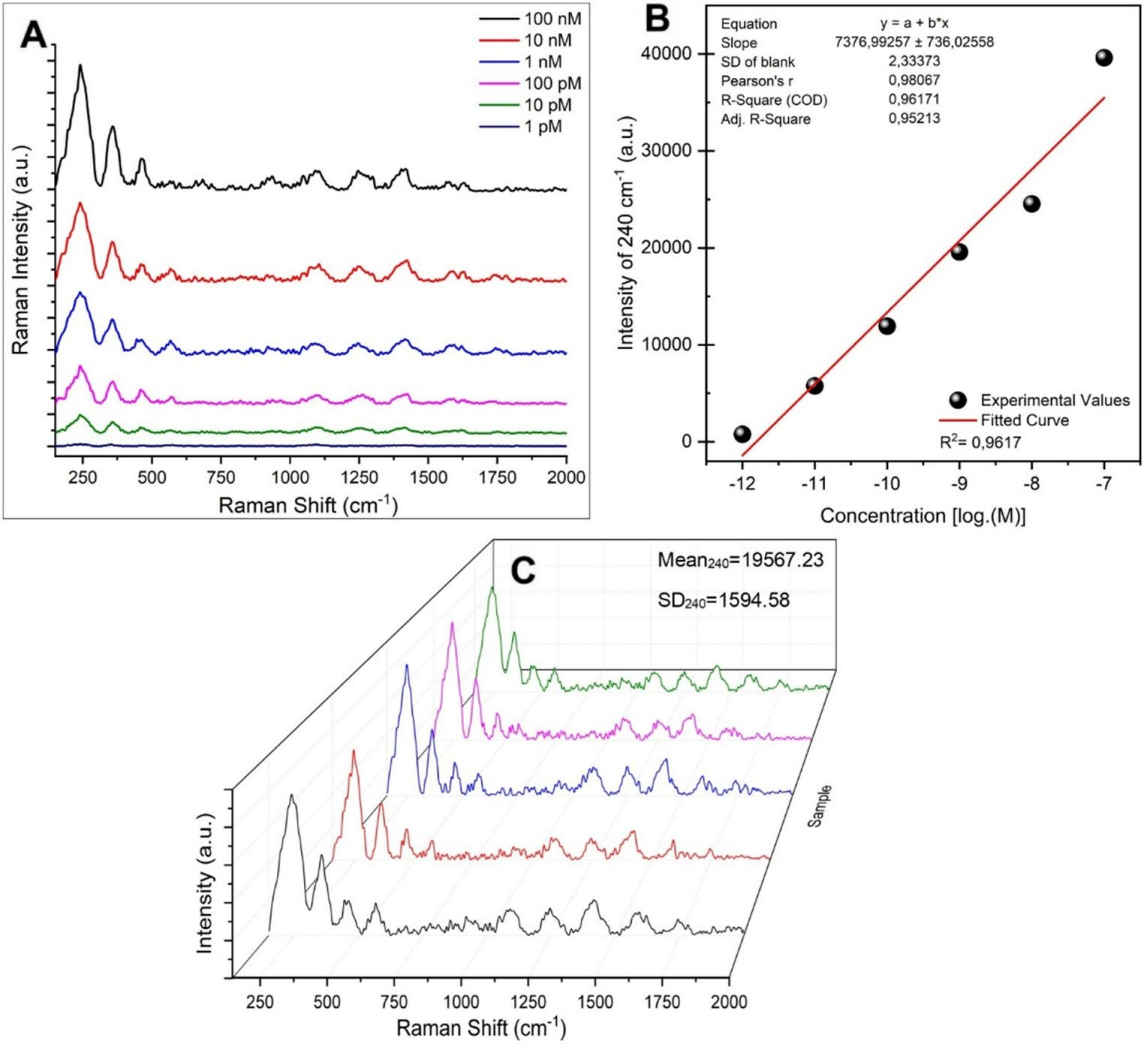
SERS analysis of ctDNA detection and reproducibility assessment. (**A**) Concentration-dependent Raman spectra of ctDNA ranging from 100 nM to 1 pM. The X-axis ranges from 250 to 200 cm⁻¹to include the 240 cm⁻¹ Au–S stretching mode used as the primary quantification band. (**B**) Calibration plot correlating Raman intensity at 240 cm⁻¹ (averaged across ten randomly selected measurement positions per substrate) with the logarithm of ctDNA concentration (R² = 0.9617). (**C**) Three-dimensional spectral plots obtained from five independent SERS substrates, each spectrum representing the mean of ten measurement positions (The calculated mean and standard deviation of the 240 cm⁻¹ band (Mean₂₄₀ = 19567.23, SD₂₄₀ = 1594.58) demonstrate the inter-substrate reproducibility of the detection platform)

To confirm the molecular fingerprint of the fluorescence findings, the LCR-amplified products were analyzed by SERS on nanostructured OnSpec-Lite substrates. Concentration-dependent Raman spectra were acquired across a ctDNA range of 100 nM to 1 pM; the spectral acquisition window was set to begin at ~ 200 cm⁻¹ to fully capture the 240 cm⁻¹ Au–S stretching mode selected as the primary quantification band (Fig. 4A). Prior to intensity extraction, all raw Raman spectra were processed using JASCO SpectraManager II software according to the following standardized pipeline: (i) automatic baseline correction (auto baseline fitting) within the 200–2000 cm⁻¹ acquisition range; (ii) fluorescence background removal using circle-type fitting with an approximation value of ~ 500, selected to adequately suppress the broad photoluminescence background without distorting the Raman bands of interest; (iii) noise reduction by Fast Fourier Transform (FFT) filtering; (iv) spectral smoothing by the means-movement method; and (v) zero-baseline correction to set the spectral baseline to zero prior to plotting. These preprocessing steps were applied identically and consistently to all sample and control spectra. The calibration curve (Fig. 4B) was constructed by plotting the mean 240 cm⁻¹ intensity—averaged across 10 randomly selected measurement positions per substrate—against the logarithm of ctDNA concentration; error bars represent the standard deviation (SD) of those 10 measurements. A strong linear correlation (R² = 0.9617) was observed, confirming the quantitative reliability and reproducibility of the SERS readout across the tested concentration range.

Typical nucleic-acid SERS signatures generally appear at higher wavenumbers (e.g., 728 cm^− 1^ for adenine, 1076 cm^− 1^ for the PO2- backbone, and 1337 cm⁻¹ for cytosine/guanine). However, we selected the 240 cm-1 feature as the primary quantitative reference. This peak is attributed to the metal – thiol (Au – S) stretching mode [27, 28], representing the specific interfacial vibrational interaction between the thiolated DNA and our nanostructured gold substrate. Utilizing this low-wavenumber mode provides a direct measure of surface-bound analyte density, independent of broader nucleobase vibrational modes. Three-dimensional spectral mapping (Fig. 4C) illustrates the overall spectral patterns; notably, each spectrum represents the mean Raman intensity obtained from ten randomly selected measurement positions across the sensing area, and the five spectra correspond to five independent SERS substrates (at 1 nM), allowing assessment of inter-substrate reproducibility of the sensing platform. In this context, the mean and standard deviation of the 240 cm⁻¹ band reflect the variability across substrates, confirming the platform’s reproducibility. As shown in Fig. 4C, the spectra from the five independent substrates exhibit consistent peak profiles and stable intensities at 240 cm⁻¹, demonstrating excellent inter-substrate reproducibility of the detection system.

**Fig. 5:**
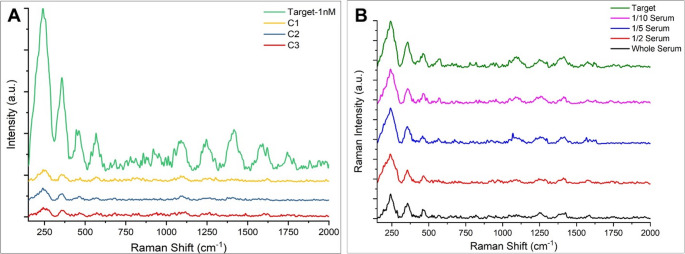
SERS analysis confirming the specificity and matrix tolerance of the optimized ctDNA detection system. (**A**) Raman spectra comparing the perfectly matched target sequence (1 nM) with single-base (C1), four-base (C2), and fully mismatched (C3) controls, demonstrating high molecular discrimination and single-base selectivity. (**B**) Raman responses obtained from ctDNA-spiked serum samples at varying dilution levels (whole, 1/2, 1/5, and 1/10)

The Raman spectral intensity decreased consistently with lowering target concentration, demonstrating strong linearity and sensitivity. Specificity was assessed using 1 nM target and three mismatched controls (C1, C2, and C3). Only the perfectly matched sequence generated prominent Raman features, whereas mismatched samples yielded substantially reduced intensity, reinforcing the assay’s molecular discrimination and single-base selectivity (Fig. 5A). The influence of serum matrix was examined by spiking ctDNA into serially diluted serum samples, as shown in Fig. 5B. Increasing the serum concentration led to a gradual reduction in Raman intensity, attributable to matrix-induced scattering and fluorescence background. Nevertheless, measurable and reproducible peaks persisted in 1/10 and 1/5 serum dilutions, indicating that moderate dilution effectively mitigated matrix effects while maintaining detection capability.

### Specificity and limit of detection

To assess the reliability and analytical performance of the developed LCR–SERS assay, feasibility tests were first conducted under optimized conditions. Fluorescence measurements of the FAM-labeled amplicons revealed strong, reproducible emission peaks, confirming efficient, consistent ligation. Extending the number of amplification cycles beyond 30 did not yield additional signal enhancement, indicating that the reaction reached saturation once all template-binding sites were occupied. This plateau behavior showed that 30 cycles achieved a practical balance between amplification efficiency and assay duration [14].

Agarose gel electrophoresis further validated these findings, displaying sharp, well-defined bands corresponding to the expected 72 bp amplicon for both mutant and wild-type targets (Fig. 2B). The relative band brightness was consistent with fluorescence intensity trends, reinforcing the reproducibility of amplification and the reliability of the optimized ligation conditions [26]. Together, these results confirmed that the selected LCR parameters were ideal for precise and efficient amplification suitable for subsequent Raman analysis.

SERS characterization of the same amplified products provided molecular-level validation of successful amplification (Fig. 4A). Characteristic Raman bands were observed near 730 cm⁻¹, 1078 cm⁻¹, and 1326 cm⁻¹, corresponding respectively to adenine ring breathing, the PO₂⁻ symmetric stretch of the DNA backbone, and cytosine/guanine ring vibrations [29, 30]. The Raman signal decreased proportionally with diminishing ctDNA concentration over the range 100 nM to 1 pM, maintaining a strong linear relationship between Raman intensity and target concentration (R² = 0.9617). The calibration curve followed y = 73.76x + 871.1, confirming excellent linearity and the system’s ability to resolve subtle variations in ctDNA levels within physiologically relevant ranges [31].

The analytical specificity of the LCR–SERS biosensing platform was subsequently evaluated by comparing Raman spectra obtained from mutant ctDNA, wild-type templates, and no-template controls (Fig. 4B). Mutant sequences exhibited distinct vibrational bands, particularly at 730 cm⁻¹ and 1326 cm⁻¹, which were either absent or significantly weaker in wild-type and control spectra. These spectral differences confirm the system’s ability to discriminate single-nucleotide variations with high precision [20, 26]. Such single-base specificity is essential for mutation-level ctDNA detection and demonstrates the molecular fidelity of the LCR ligation process when coupled with SERS-based readout [32].

Reproducibility was evaluated across five independent sensing zones on each OnSpec (L-Mark) gold substrate, demonstrating excellent uniformity, with inter-zone relative standard deviation (RSD) values below 10%. This low variation indicates high reproducibility of Raman signal intensities across different sensing areas and reflects the structural homogeneity of the NECTEC-fabricated substrate [18] (Fig. 4C). Such reproducibility is crucial for diagnostic applications, where inter-chip stability ensures analytical reliability [33, 34].

The platform’s detection performance was further evaluated using both fluorescence and SERS modalities. In fluorescence measurements (Fig. 3A), the lowest detectable ctDNA concentration above baseline was 100 pM, whereas SERS spectra acquired under identical conditions (Fig. 4A) exhibited distinguishable peaks above background, confirming comparable sensitivity between the two detection methods [14]. Therefore, the lowest experimentally validated concentrations that produced a clear signal were 100 pM for fluorescence and 1 pM for SERS. To ensure methodological clarity and reproducibility of the calculated limit of detection (LOD) and limit of quantification (LOQ), the regression slope 𝑏 used in the IUPAC equations below was derived from the linear concentration scale in the ultra-low concentration range, rather than the logarithmic scale.

$$LOD\:=\:3.3\:\times\:\:\left(\frac{\sigma\:\:}{b}\right)$$

$$LOQ\:=\:10\:\times\:\:\left(\frac{\sigma\:\:}{b}\right)$$

where σ represents the standard deviation of blank SERS signals at 240 cm^− 1^ (σ = 2.33; *n* = 10), and fluorescent signals at 475 nm wavelengths (σ = 12.49; *n* = 10). Blanks consisted of buffer-only samples processed through the entire workflows (probe immobilization, washing, and signal acquisition) under conditions identical to those of the analyte samples. The parameter b represents the slope of the linear regression from the respective calibration curves, where b = 73.77 (R^2^ = 0.9617; concentration range 1 pM – 100 nM) for SERS, and b = 69.03 (R^2^ = 0.8705; concentration range 100 pM – 1000 nM) for fluorescence [35]. Both calibrations exhibited strong linearity across their respective ranges, consistent with the earlier calibration results described above. Based on these parameters, the calculated LOD was 1.03 fM for SERS and 11.94 fM for fluorescence, and the corresponding limit of quantification was 3.12 fM and 36.17 fM, respectively (listed in Table S2). To ensure methodological clarity regarding the calculated analytical limits, it should be noted that the regression slope (b) is derived from the calibration curves plotted as signal intensity versus the logarithm of the ctDNA concentration. Consequently, the x variable in the regression equations explicitly represents log[M]. The LOD and LOQ were evaluated based on this logarithmic relationship, and the resulting logarithmic values were subsequently converted back into absolute concentrations (reported in fM) to yield the final detection and quantification limits.

These results confirm the ultrasensitive analytical capability of the developed LCR–fluorescence–SERS biosensing platform for ctDNA detection. The achieved detection limit indicates high analytical sensitivity compared with previously reported LCR- or SERS-based ctDNA biosensing strategies, which typically exhibit LODs in the 10–100 fM range [19, 36]. These results confirm the analytical sensitivity and reproducibility of the integrated detection system under the tested proof-of-concept conditions.

## Discussion

The integration of LCR, fluorescence quantification, and SERS analysis demonstrates a distinctive analytical synergy that advances the molecular detection of ctDNA. Although the readouts are acquired sequentially, both fluorescence and SERS analyses are performed on the same LCR-amplified products, forming a unified analytical workflow rather than independent experiments. Our tri-modular design bridges the selectivity of enzymatic ligation with the high-fidelity spectral confirmation of nanoplasmonic sensing by analyzing the same amplified products in both approaches. This platform therefore provides a multidimensional readout that ensures analytical robustness and confidence in single-base mutation discrimination [10, 26]. In this framework, fluorescence reporting allows real-time monitoring of reaction efficiency, while the SERS module provides orthogonal validation through the molecular vibrational fingerprint of the amplified target [19, 37]. Together, both methods offer a built-in validation mechanism to minimize false positives and provide cross-validated results that are highly valuable for high-stakes diagnostics.

Compared with conventional polymerase-based approaches such as qPCR and dPCR, which rely on primer extension and are susceptible to nonspecific amplification or misincorporation events, the ligase-mediated reaction in LCR offers an intrinsic biochemical safeguard. The thermostable ligase used here catalyzes phosphodiester bond formation only when complete complementarity exists between the probe and target strands, yielding exceptional single-nucleotide specificity—an indispensable feature for liquid biopsy applications where mutant alleles exist at extremely low abundance [38, 39].

The integrated fluorescence–SERS biosensing platform demonstrated strong agreement between fluorescence quantification and Raman-based molecular validation. Under the optimized conditions “30 cycles, 60°C ligation temperature, 2 µL enzyme, and a 1-min ligation period,” the system achieved high reproducibility with minimal background noise (Fig. 2). The lowest experimentally validated ctDNA concentration was 100 pM and 1 pM for fluorescence and SERS modalities, respectively; the calculated LOD derived from linear regression of the SERS calibration curve was 1.03 fM (LOQ 3.12 fM), equivalent to ~ 0.0245 pg mL⁻¹ (LOQ ~ 0.0741 pg mL⁻¹) or ~ 6.20 × 10² copies µL⁻¹ (LOQ ~ 1.88 × 10³ copies µL⁻¹) for the 72-nt target [26]. Meanwhile, the fluorescence modality yielded a calculated LOD of 11.94 fM (LOQ 36.17 fM), which corresponds to ~ 0.2842 pg mL⁻¹ (LOQ ~ 0.8608 pg mL⁻¹) or ~ 7.19 × 10³ copies µL⁻¹ (LOQ ~ 2.18 × 10⁴ copies µL⁻¹).

This analytical performance demonstrates the potential advantages of coupling ligation-based amplification with plasmonic signal enhancement for sensitive ctDNA detection. The combined strategy bridges quantitative sensitivity with molecular specificity, establishing a robust framework for mutation detection in liquid biopsy and other precision diagnostic applications [3].

The nanostructured OnSpec (L-Mark) substrate, fabricated by NECTEC, contributes to the assay’s sensitivity and reproducibility, consistent with ongoing developments in SERS-based liquid biopsy technologies aimed at improving spectral stability and analytical reliability. It’s precisely engineered plasmonic features generated reproducible electromagnetic “hot spots,” producing highly consistent SERS enhancement across all sensing zones. The inter-zone relative standard deviation (RSD < 10%) attests to the optical stability and surface homogeneity of this substrate, aligning well with previously reported performance of high precision nanoplasmonic chips [15]. Importantly, integrating fluorescence and SERS modes created an internal verification mechanism: fluorescence provided rapid, quantitative feedback, while SERS provided molecular-level identity confirmation. This dual validation minimizes error propagation and strengthens diagnostic reliability [26, 32].

A particularly noteworthy outcome is the calculated detection limit of 1.03 fM, derived from the calibration equation y = 73.77x + 871.1 (R² = 0.9617), while the lowest experimentally validated concentration detected by SERS was 1 pM. This calculated detection is comparable to, and in several cases lower than, those reported for previously described LCR- or SERS-based ctDNA biosensors, which typically range from 10 to 100 fM under their respective assay conditions [33]; however, direct numerical comparison should be interpreted cautiously given the heterogeneous assay matrices, target formats, and analytical workflows across studies. The integration of LCR amplification with dual optical readout within a single workflow promises an analytical strategy toward high-fidelity molecular diagnostics [26].

In complex matrices such as diluted serum, the platform maintained excellent signal reproducibility and base-mismatch discrimination, despite moderate attenuation of fluorescence and Raman signals caused by autofluorescence and scattering. This robustness validates the feasibility of the proposed workflow in controlled serum-based environments; however, additional validation using authentic clinical plasma samples will be necessary before translational application can be considered [3].

Compared with other nanoplasmonic biosensing strategies, the present system distinguishes itself by combining analytical rigor with operational simplicity [36, 37]. It requires fewer enzymatic components, eliminates complex amplification cascades, and achieves sensitivity comparable to or superior to that of more intricate methods such as exonuclease-assisted cyclic cleavage (LOD ≈ 9.1 fM) [26]. Collectively, these outcomes establish the LCR–fluorescence–SERS configuration as an efficient, reliable, and high-performance analytical platform for ctDNA quantification and mutation-level differentiation (Table 2). However, direct comparison of limits of detection across different ctDNA sensing platforms should be interpreted cautiously, as assay matrices, extraction procedures, target formats, and analytical workflows vary significantly between studies, which can influence the reported analytical sensitivity. It is important to note that the current platform represents a highly sensitive analytical proof-of-concept evaluated under controlled conditions rather than a clinical assay. While we have successfully demonstrated controlled analytical performance, selective single-nucleotide discrimination, and dual-readout feasibility, extensive future work remains necessary before translational investigations can be performed. Future work would therefore include validation in original patient plasma to fully assess pre-analytical variability, expansion to broader multiplexed panels, and rigorous testing across multiple clinical examinations [16, 37].

**Table 2:** Comparison of the analytical performance of the present LCR–fluorescence–SERS

Target & Context	Target Type	Matrix	Detection Principle	Signal Readout	Calculated Sensitivity (LOD)	Tested Linear Range	Sample Volume	Assay Time	Real Sample Validation	Multiplex Capability	Ref
Nucleic acids (model target; RCA-SERS coupling)	Nucleic acid target (model system)	Not specified in abstract-level summary (likely buffer)	Rolling circle amplification (RCA) + SERS	SERS of reporter bound to RCA products	LOD ≈ 10.0 pM (reported as achievable)	NR	NR	NR	NR	No	[40]
CRC plasma ctDNA mutations (KRAS G12V, KRAS G13D, BRAF V600E)	Allele-specific PCR amplicons	Plasma ctDNA (plus CRC cell lines)	Multiplex PCR + multicolor SERS nanotags + magnetic bead enrichment	Label-based SERS nanotag spectra (portable Raman)	0.1% mutant allele fraction detectable (from WT cfDNA background)	NR	NR	NR	Yes (blinded CRC plasma testing; validated by ddPCR)	3-plex	[19]
Melanoma ctDNA mutations (BRAF V600E, c-Kit L576P, NRAS Q61K)	Multiplex PCR amplicons	Plasma-derived ctDNA	Single-tube multiplex PCR + SERS nanotags	Label-based SERS nanotags	NR (reported 0.1% mutant allele fraction, 10/10,000 copies)	NR	NR	NR	Yes (patient ctDNA vs. ddPCR)	3-plex	[20]
Lung cancer ctDNA (KRAS G12D), incl. patient serum	Mutation-selective ctDNA target	PBS; FBS; human physiological media; **patient serum**	RNase HII-mediated target recycling; “frequency shift” assay	Enzymatically amplified SERS frequency-shift readout	“Sub-fM” in PBS (numeric NR)	NR	NR	NR	Patient serum detection claimed	Single-plex	[41]
DIPG-related ctDNA (H3.3 point mutation), spiked human samples	Mutation-selective ctDNA target	PBS + spiked human serum	Exonuclease III cyclic enzymatic amplification + SERS substrate	Label-based SERS (Cy5); quantity at 1366 cm^− 1^	7.9 fM (PBS), 9.1 fM (serum)	NR	NR	2 h incubation at 37 °C (total NR)	Spiked human donor sample extraction + analysis	Single-plex	[26]
Glioma-related ctDNA mutations (IDH1 R132H, BRAF V600E) in mouse model	Mutation-selective ctDNA targets	Mouse serum (PBS also mentioned)	CHA amplification + magnetic aggregation (Au–Ag nanoshuttles)	Label-based SERS; linear vs. log$\:{}_{10}$[C]	**6.01** aM (IDH1), 5.48 aM (BRAF) (serum)	10 aM–100 pM (fit vs. logC; points listed)	NR	NR	Mause serum application	2-plex	[42]
72-nt mutant ctDNA (KRAS locus)	Synthetic 72-nt ctDNA fragment	Buffer; diluted serum (1/2, 1/5, 1/10)	LCR + dual-mode fluorescence/SERS	Fluorescence (FAM–AuMNP) + SERS (OnSpec-Lite chip)	LOD = 11.94 fM, LOQ = 3.12 fM (Fluorescent) LOD = 1.03 fM, LOQ = 36.17 fM (SERS)	Fluorescence: 1000 nM–100 pM; SERS: 100 nM–1 pM	20 µL (LCR) + 10 µL (on SERS chip)	~ 1.5–2 h total (30 cycles + hybridization + SERS prep)	Diluted serum 1/2, 1/5, 1/10	No	This work

The developed LCR–Fluorescence–SERS biosensor demonstrated superior femtomolar-level sensitivity compared with other enzymatic and plasmonic ctDNA detection methods, while maintaining high specificity and reproducibility. The integration of LCR amplification and SERS fingerprinting enabled both quantitative and confirmatory molecular recognition within a single workflow.

## Conclusion

To the best of our knowledge, this study presents an integrated LCR–fluorescence–SERS biosensing workflow optimized for sensitive ctDNA mutation detection with single-nucleotide resolution. The platform merges the molecular precision of enzymatic ligation with the quantitative verification of nanoplasmonic spectroscopy, forming a unified workflow that converts molecular recognition into reproducible optical signals. Under optimized reaction conditions **(**60 °C ligation temperature, 30 cycles, and 2 µL of T4 DNA ligase) the system achieved a calculated LOD of 1.03 fM and a LOQ of 3.12 fM while the lowest experimentally detectable ctDNA concentration was 1 pM.

Beyond numerical performance, the assay demonstrated robust reproducibility (RSD < 10%), operational simplicity, and adaptability to serum-based environments, indicating its potential for future development of liquid-biopsy diagnostic platforms. The ability to simultaneously obtain real-time fluorescence quantification and confirmatory SERS fingerprints minimize false positives and ensures molecular accuracy, strengthening the analytical reliability of mutation detection within controlled experimental conditions.

Furthermore, the platform’s modular design permits rapid reconfiguration of probe sequences for diverse oncogenic mutations, including EGFR (T790M), KRAS (G12D), and PIK3CA (E545K), supporting future development of multiplexed or personalized ctDNA panels. In essence, this LCR–fluorescence–SERS biosensor embodies a new generation of dual-modality molecular diagnostics, providing a promising analytical foundation for future translational investigations. Its combination of ultra sensitivity, reliability, and versatility positions it as a promising analytical proof-of-concept for early cancer detection, therapeutic monitoring, and precision oncology applications.

## Supplementary Information

Below is the link to the electronic supplementary material.

Supplementary Material 1 (DOCX 6.33 MB)

## Data Availability

No datasets were generated or analysed during the current study.

## Declarations

### Generative AI in scientific writing

During the preparation of this work, the authors used the Gemini AI program in order to check the grammar and improve the English writing of the manuscript. After using this tool/service, the authors reviewed and edited the content as needed and take full responsibility for the publication’s content.
