# Supplementary material for: Development of an ligase chain reaction–fluorescence–SERS multimodal biosensing workflow for ultrasensitive detection and single-base discrimination of KRAS ctDNA
Source: Mikrochim Acta. 2026 Jun 13;193(7):470. doi: 10.1007/s00604-026-08168-3 (PMC13264572; doi:10.1007/s00604-026-08168-3)
Supplement: Supplementary file 1 — Supplementary Material 1 (DOCX 6.33 MB) [file 604_2026_8168_MOESM1_ESM.docx]

**Supporting Information**

**Development of an ligase chain reaction–fluorescence–SERS multimodal biosensing workflow for ultrasensitive detection and single-base discrimination of *KRAS* ctDNA**

Sohila Mostafa¹, Dilek Kanarya^1^, Khwanchai Tantiwanichapan*^2^*, Raju Botta*^2^*, Pitak Eiamchai*^2^*,
Pacharamon Somboonsaksri*^2^*, Nimet Yıldırım Tirgil^1,3*^

*^1^ Department of Biomedical Engineering, Ankara Yıldırım Beyazıt University, Ankara, Türkiye*

*^2^ National Electronics and Computer Technology Center (NECTEC), National Science and Technology Development Agency (NSTDA), 112, Khlong Nueng, Khlong Luang, Pathum Thani, 12120, Thailand.*

*^3^ Department of Metallurgy and Materials Engineering, Ankara Yıldırım Beyazıt University, Ankara, Türkiye.*

***** Ayvalı Mh. Takdir Cad.150 Sk. No:5 Etlik-Kecioren/Ankara 06010, Türkiye.

E-Mail: nyildirim.tirgil@aybu.edu.tr


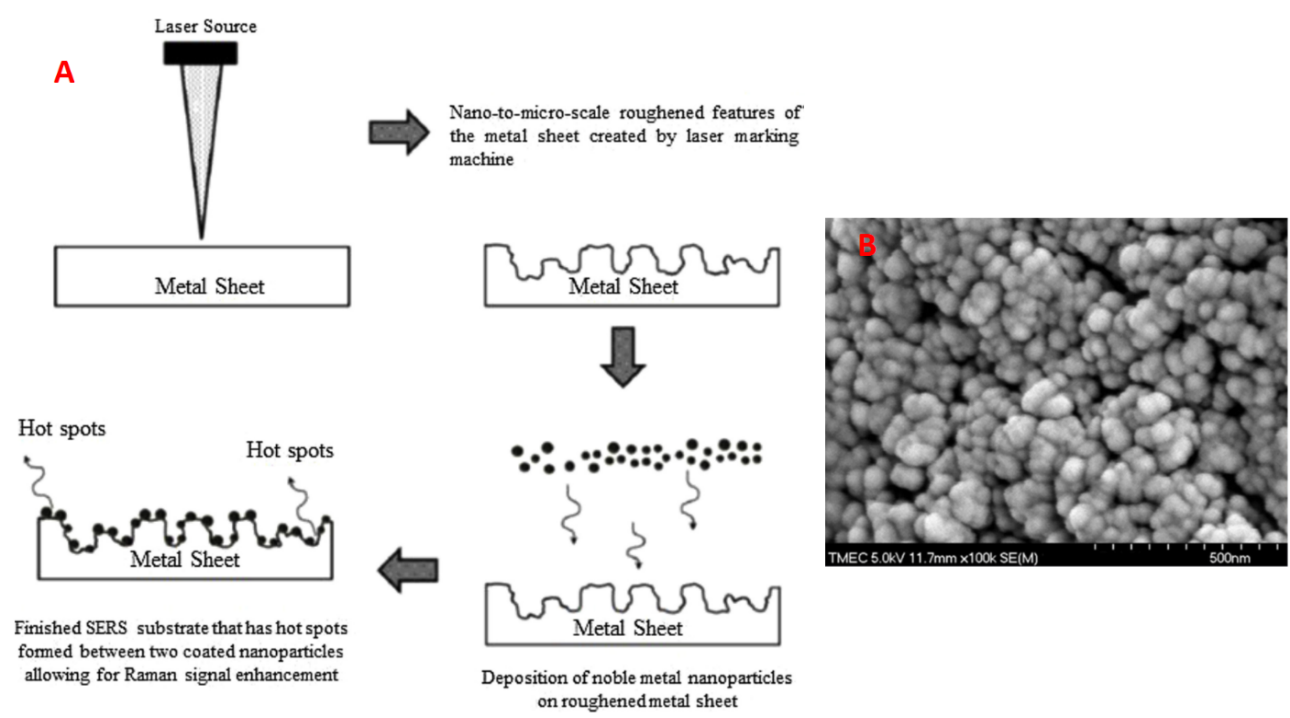


**Figure S1.** Fabrication and characterization of the Al/Au SERS Sensors. (A) Schematic of the SERS chip substrate production process: creation of a micro-to-nano scale rough texture on a metal sheet surface using a laser marking machine, followed by the deposition of metal nanoparticles onto the roughened surface. (B) FESEM micrograph showing gold nanostructures grown within the laser-etched pits on an aluminum surface (Adapted from Botta et al. 2020).


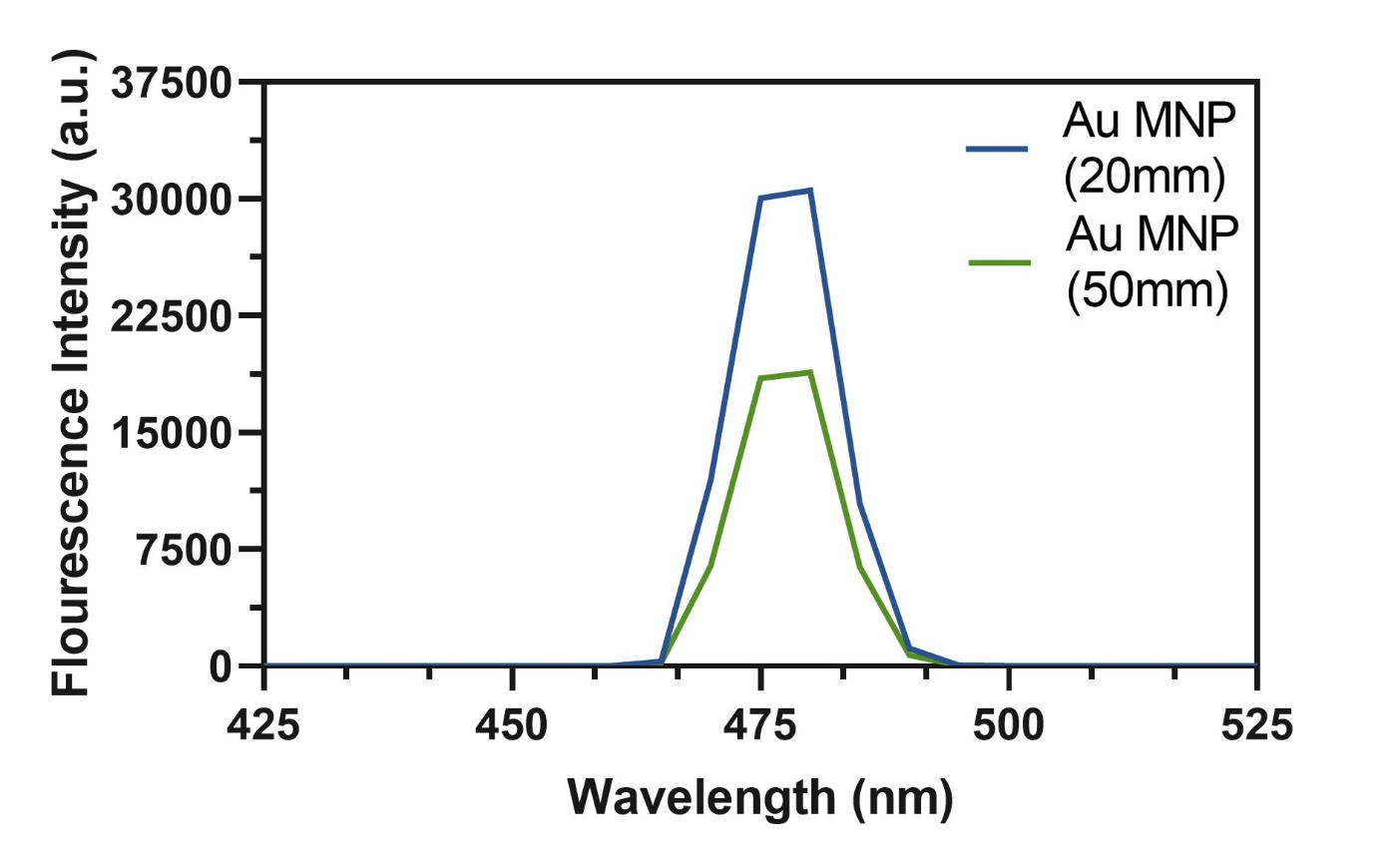


**Figure S2**: Comparison of fluorescence emission spectra of Au MNP conjugates with different particle sizes (20 nm and 50 nm) functionalized with thiolated primers. The Au MNP (20 nm) conjugates exhibited higher fluorescence intensity than the 50 nm counterparts, indicating improved particle dispersion and reduced quenching effects, thereby enhancing overall signal sensitivity.

**Table S1:** Optimized parameters for the LCR assay**.**

| **Parameters** | **Tested Conditions** | **Optimized Condition** | **Remarks** |
| --- | --- | --- | --- |
| Temperature (°C) | 53, 60, 67 | 60 | Balanced hybridization and ligation efficiency |
| Ligase Volume (µL) | 1, 2, 3 | 2 | Maximal signal without background rise |
| Cycle Number | 30, 40, 50 | 30 | Stable amplification, minimal nonspecific ligation |
| Incubation Time (min) | 1,3 | 1 | Sufficient for complete ligation |
| DNA Concentration | 100 pM to 1000 nM (Fluorescent)  1 pM to 1000 nM (SERS) | LOD = 11.94 fM, LOQ = 3.12 fM (Fluorescent)  LOD = 1.03 fM, LOQ = 36.17 fM (SERS) | Experimental detected Linear range and calculated LOD/LOQ for fluorescence/SERS response |
| Serum Dilution | Whole, 1/2, 1/5, 1/10 | 1/5 | Reduced matrix interference |
| Primer Ratio | 1:1, 1:2, 2:1 | 1:1 | Optimal ligation yield |
| Reaction Volume (µL) | 10, 20, 30 | 20 | Consistent temperature control |

**Table S2:** Analytical performance parameters of the dual-modality LCR-fluorescence-SERS biosensor for ctDNA detection.

| **Parameter** | **Fluorescence** | **SERS** |
| --- | --- | --- |
| **Slope, b** | 3452.18 | 7376.99 |
| **SD (σ)** | 12.49 | 2.33 |
| **R^2^** | 0.8705 | 0.9617 |
| **Calculated LOD** | 11.94 fM | 1.03 fM |
| **Calculated LOQ** | 36.17 fM | 3.12 fM |
| **Experimentally Tested Linear Range** | 100 pM – 1000 nM | 1 pM – 100 nM |
